# The Role of the Gastrointestinal Microbiota in Parkinson’s Disease

**DOI:** 10.3390/biom15010026

**Published:** 2024-12-28

**Authors:** Maurizio Gabrielli, Lorenzo Zileri Dal Verme, Maria Assunta Zocco, Enrico Celestino Nista, Veronica Ojetti, Antonio Gasbarrini

**Affiliations:** 1 Department of Medical and Surgical Sciences, Fondazione Policlinico Universitario A. Gemelli IRCCS, Università Cattolica del Sacro Cuore, 00168 Rome, Italy; lorenzo.zileridalverme@policlinicogemelli.it (L.Z.D.V.); mariaassunta.zocco@policlinicogemelli.it (M.A.Z.); enricocelestino.nista@policlinicogemelli.it (E.C.N.); antonio.gasbarrini@unicatt.it (A.G.); 2 Internal Medicine Department, San Carlo di Nancy Hospital, Università UniCamillus, 00131 Rome, Italy; veronica.ojetti@unicamillus.org

**Keywords:** Parkinson’s disease, brain–gut axis, microbiota, *Helicobacter pylori*, small-intestinal bacterial overgrowth, probiotics, prebiotics, fecal microbiota transplantation, synbiotics, rifaximin

## Abstract

Background/Objectives: Parkinson’s disease (PD) is a progressive neurodegenerative disorder characterized by the loss of dopaminergic neurons leading to debilitating motor and non-motor symptoms. Beyond its well-known neurological features, emerging evidence underscores the pivotal role of the gut–brain axis and gastrointestinal microbiota in PD pathogenesis. Dysbiosis has been strongly linked to PD and is associated with increased intestinal permeability, chronic inflammation, and the production of neurotoxic metabolites that may exacerbate neuronal damage. Methods: This review delves into the complex interplay between PD and dysbiosis, shedding light on two peculiar subsets of dysbiosis, *Helicobacter pylori* infection and small-intestinal bacterial overgrowth. These conditions may not only contribute to PD progression but also influence therapeutic responses such as L-dopa efficacy. Conclusions: The potential to modulate gut microbiota through probiotics, prebiotics, and synbiotics; fecal microbiota transplantation; and antibiotics represents a promising frontier for innovative PD treatments. Despite this potential, the current evidence is limited by small sample sizes and methodological variability across studies. Rigorous, large-scale, randomized placebo-controlled trials with standardized treatments in terms of composition, dosage, and duration are urgently needed to validate these findings and pave the way for microbiota-based therapeutic strategies in PD management.

## 1. Introduction

PD is a common neurological disorder affecting approximately 1–2% of individuals over 65 years old. Its prevalence is projected to rise in the coming years as age is a significant risk factor [1]. PD is characterized by motor dysfunction with classic symptoms such as tremor, rigidity, postural instability, and bradykinesia. These symptoms appear when most of the dopaminergic neurons in the substantia nigra pars compacta of the CNS are damaged [2]. Apart from neurological motor impairment, PD can cause neurovegetative dysfunctions in all systems, including the digestive one, where disorders appear more evident. GI symptoms, particularly constipation, are prevalent in most PD patients and often precede the onset of motor symptoms by several years [3].

The etiopathogenesis of PD remains unclear. α-synuclein, a synaptic protein involved in regulating neuronal functions like mitochondrial activity and axonal transport, is implicated in the disease. In idiopathic PD (approximately 90% of cases), misfolded α-synuclein accumulates and forms insoluble clumps known as Lewy bodies. These protein aggregates accumulate in neurons of both the ENS and the CNS [2,3]. Microglia, the most abundant innate immune cells in the CNS, play a crucial role in maintaining brain homeostasis. These resident macrophages sense environmental changes, remove cellular debris, and provide neurotrophins, interacting closely with nearby neurons [4]. In PD, misfolded α-synuclein can trigger excessive microglial activation. Activated microglia migrate to sites of injury, modulating inflammation and phagocytosis. Chronic neuroinflammation may further exacerbate neuronal damage and disease progression [5].

α-synuclein aggregates are present in the submucosal and myenteric plexuses of the ENS long before they appear in the brain [3]. Building on the topographic distribution of Lewy bodies observed in PD patients, Braak et al. proposed in 2003 that a pathogen may enter the GI tract, damage neurons, and trigger α-synuclein protein aggregation. This aggregation then propagates from the GI tract to the CNS and higher cortical regions via the vagus nerve [6,7]. In 2013, Holmqvist et al. provided experimental evidence supporting this “prion-like” spread. Aggregated α-synuclein injected into the gut in healthy rats was transported from the intestine, along the vagus nerve, to the brain [8].

In recent years, the bidirectional communication between the CNS and GI system has been extensively characterized, leading to the concept of the “gut-brain axis”. Moreover, emerging evidence highlights the significant role of the GI microbiota in modulating this interaction, both in health and in diseases such as PD. Consequently, the term “microbiota-gut-brain axis” is now preferred [9].

By critically reviewing the current literature, this review aims to provide a comprehensive overview of the role of the GI microbiota and the therapeutic potential of targeting GI dysbiosis in PD.

## 2. Methods

We conducted a comprehensive literature review of English-language articles published between January 2000 and November 2024. We searched the PubMed, MEDLINE, and Cochrane databases using the following Medical Subject Headings: “Parkinson’s Disease” OR “Neurological” OR “Neurodegenerative” AND “Gut Microbiota”, “Gastrointestinal Microbiota”, “Brain-gut axis”, “Probiotic”, “Prebiotic”, “Synbiotic”, “Antibiotic”, “Fecal Microbiota Transplantation”, “Helicobacter pylori”, “small intestine bacterial overgrowth”, and “SIBO”. We also manually searched the reference lists of included articles.

We excluded book chapters, conference annals, case reports, pediatric studies, and articles without full-text availability. The final selection of articles was determined through a consensus discussion. From an initial identification of 7582 articles, we finally included a total of 77 relevant papers in the present review (41 in the dysbiosis section, 20 in the HP section, and 16 in the SIBO section).

## 3. Brain–Gut Axis

This bidirectional communication between the GI system and CNS is mediated by a complex interplay of neural, hormonal, and immunological pathways. The human GI tract is innervated by all three divisions of the autonomic nervous system: the ENS, the sympathetic nervous system, and the parasympathetic nervous system.

The ENS, composed of approximately 600 million neurons, forms a complex network of ganglia-rich nerve connections within the GI tract walls, extending from the esophagus to the anal canal [10]. Among its many functions, the ENS regulates peristaltic movement, secretion, and immunological responses. Additionally, it collaborates with peripheral glial cells (enteric glia) to maintain epithelial barrier integrity and modulate inflammation [10]. While the ENS, along with the intestinal endocrine system, can independently regulate primary GI functions, it is bidirectionally interconnected with the CNS, primarily via the parasympathetic nervous system (particularly the vagus nerve) but also through the sympathetic nervous system. These systems form complex relationships with the ENS, with their fibers penetrating the GI tract wall and establishing synaptic connections with ENS neurons [10].

The vagus nerve is the most significant direct bidirectional communication pathway between the GI tract and the CNS. It also interacts with extraintestinal hormonal pathways, notably influencing the hypothalamic–pituitary–adrenal axis, a major player in stress response [9]. Interestingly, truncal vagotomy has been associated with a nearly 50% reduction in PD risk, and in a rat model, this intervention halted the transneuronal propagation of injected α-synuclein from the gut to the CNS [11,12].

## 4. From the Brain–Gut Axis to the Microbiota–Brain–Gut Axis

The human GI tract harbors a complex and dynamic ecosystem, primarily composed of bacteria, along with fungi, viruses, and protozoa. Weighing approximately 1.5 kg and comprising around 100 trillion microorganisms, this microbiota is essential for human health [13,14]. Immediately after birth, the nearly sterile human gut is colonized by the mother’s microbiota, with the mode of delivery significantly influencing the initial microbial composition. Subsequent factors like breastfeeding, the diet, and environmental exposure further shape the gut microbiome [13,14]. In adulthood, the microbiome remains relatively stable, though it can be influenced by external factors such as the diet, medications, illnesses, and stress.

Humans and their microbiota exist in a symbiotic relationship, with humans providing nutrients and the microbiota performing essential functions, including the digestion of complex carbohydrates, the production of SCFAs, vitamin synthesis, immune system regulation, protection against pathogens, drug and toxin metabolism, the maintenance of intestinal barrier function, the regulation of metabolism, energy balance, and inflammation [13,14].

A “healthy” GI microbiota is characterized by high diversity, with a wide variety of microbial species and abundant populations [13,14]. This diversity, particularly the balance between beneficial microorganisms and the host environment, is a key indicator of gut health and is closely linked to ecosystem stability and positive health outcomes [14,15].

The GI microbiota plays a crucial role in modulating the gut–brain axis, leading to the concept of the microbiota–gut–brain axis. The microbiota influences brain function through various pathways, both locally in the GI tract and via the circulatory system. The microflora interacts with the immune system, neuroendocrine cells, the ENS, and the parasympathetic and sympathetic nervous systems through the production of molecules like neurotransmitters, metabolites, and hormones [9,16]. The microbiota can directly synthesize or stimulate the production of neurotransmitters such as serotonin, GABA, dopamine, glutamate, melatonin, and acetylcholine. Additionally, several substances produced by microorganisms, including indole derivatives, SCFAs, branched-chain amino acids, and vitamins, influence the gut–brain axis. The GI microflora also affects the secretion of hormones like ghrelin, insulin, nestin, cholecystokinin, and leptin [9,16]. The interaction between the GI microbiota and the immune system is also critical. The microflora is involved in the maturation and function of innate and adaptive immunity through a continuous dialogue based on various pathways. The microbiota interacts with TLRs on immune cells, promoting the maturation of dendritic cells and the production of cytokines essential for immune homeostasis. SCFAs and other metabolites produced by the microbiota influence the differentiation and function of T cells, modulating immune responses [17]. SCFAs are also essential for the integrity of the intestinal barrier, a crucial defense mechanism against pathogens and toxins [18].

## 5. The Role of Dysbiosis in PD

A disruption of the physiological balance in the GI tract’s microbial population, characterized by both qualitative and quantitative shifts, is termed “dysbiosis”. This generic definition encompasses two specific forms of GI dysbiosis, which deserve a separate discussion. These are gastric colonization by HP, which disrupts the normal gastric microbiota in approximately half of the world’s population [19], and the equally prevalent SIBO [20,21].

Dysbiosis is linked to various intestinal and extra-intestinal diseases. In recent years, numerous studies have explored the role of alterations in the GI microbiota in PD.

### 5.1. Gut Microbiota Dysbiosis in PD

Although a consensus on the ideal composition of a healthy human GI microbiota remains elusive, numerous studies have identified substantial modifications in the intestinal microbial colonies of PD patients.

Bai et al. recently published a systematic review and subgroup meta-analysis of 14 studies (1045 PD cases and 821 healthy controls) assessing characteristics of the gut microbiota by raw 16S rRNA gene sequences. They showed significant differences in microbial abundance between PD patients and controls. Specifically, *Lachnospiraceae*, *Prevotellaceae*, *Erysipelotrichaceae*, and *Faecalibacterium* were significantly less abundant in PD patients while *Bifidobacteriaceae*, *Rikenellaceae*, *Ruminococcaceae*, *Lactobacillaceae*, *Verrucomicrobiaceae*, and *Christensenellaceae* counts were significantly increased [22]. The alteration of the microbiota observed in these studies may have an etiopathogenetic role in PD. *Lachnospiraceae* is able to produce butyrate from the fermentation of dietary fibers in the gut. The SCFA butyrate plays an important role in the trophism of intestinal cells and in the production of the mucous layer. Reduced SCFAs are associated with intestinal epithelial barrier dysfunction [23]. The consequent increased intestinal permeability, commonly observed in PD patients [18,24], exposes the intestinal nerve plexus to various toxins, which may favor the abnormal aggregation of α-synuclein fibrils [25,26]. Additionally, toxins and products of inflammation from the GI tract wall can enter the systemic circulation and reach the brain. It has been documented that increasing degrees and durations of systemic inflammation can lead to a more permeable blood–brain barrier. The subsequent passage of toxins, immune cells or antibodies, and antigens can activate microglia, which are responsible for neuroinflammation [27]. SCFA deficiency in the gut lumen could also be associated with neuroinflammation due to their involvement in immunotolerance by promoting the differentiation of naïve T cells into regulatory T cells and regulating macrophage polarization [18,23]. Reductions in *Prevotellaceae* levels lead to a decrease in the secretion of ghrelin, a hormone produced by enteroendocrine cells of the GI tract that, among other functions, activates dopaminergic neurons and is involved in neuroprotection [22,28]. Interestingly, since *Bifidobacteriaceae* species catabolize L-dopa, the abundance of this family is associated with the need to administer higher doses of the drug [29]. *Akkermansia* spp., belonging to the family of *Verrucomicrobiaceae*, are commonly present in the human intestinal microbiota. An abundance of *Akkermansia muciniphila* has been associated with beneficial effects on health, leading to its use as a probiotic. It has been shown to promote the integrity of the epithelial cell layer and positively modulate the immune system [30,31]. However, studies have also linked an abundance of *Akkermansia* spp. to an increased risk of neurological diseases. The mechanism by which *Akkermansia* can exert beneficial or harmful effects on health may depend on various factors, including the host’s genetic composition, the immunomodulatory properties of the strain, interactions with other members of the GI microbiota, and drug interactions [30,31]. Increased *Akkermansia* counts in PD may constitute a consequence of constipation as constipated individuals often have a gut microbiome enriched in this bacterium [32]. This can lead to a depleted intestinal mucus layer, drier stools, reduced goblet cell numbers, and impaired intestinal barrier function, particularly in the presence of GI dysbiosis. Certain strains may dysregulate the immune system and promote inflammation [30,31]. Additionally, *Akkermansia muciniphila* may increase calcium uptake at the mitochondrial level, leading to the generation of reactive oxygen species and aggregation of α-synuclein in the ENS [33].

A recent metagenomic case-control analysis by Metcalfe-Roach et al. reported a large-scale alteration of the gut microbiota in PD patients [34]. As observed in previous studies, the gut microbiota was more fragmented in PD compared to controls, with a substantial loss of intermicrobial connectivity. The increased transit time typical of PD may have contributed to this finding [35]. Bacterial species were differentially abundant in PD compared to controls. *Alistipes indistinctus*, *Blautia obeum*, *Coprococcus catus*, and *Ruthenibacterium lactatiformans* were associated with PD while *Blautia wexlerae*, *Roseburia intestinalis*, and *Roseburia inulinivorans* were control-associated. These results aligned with previous metagenomic studies on PD, highlighting the consistency of major microbial changes across various demographic groups [36,37,38]. The microbial functions of PD and controls were also functionally distinct. As observed in other cohorts, genes related to carbohydrate transport and metabolism were moderately depleted in PD [38]. This may have led to the impaired hepatic detoxification of toxic metabolites such as p-cresol and pesticides, known risk factors for PD [39]. Increased glutaryl-CoA degradation and glutamate synthase are particularly associated with progression, suggesting that the dysregulation of microbial glutamate production may affect brain health [40]. Altered purine metabolism by PD-associated dysbiosis may contribute to the higher serum purine levels observed in PD and associated with PD progression [41,42].

**Figure 1:**
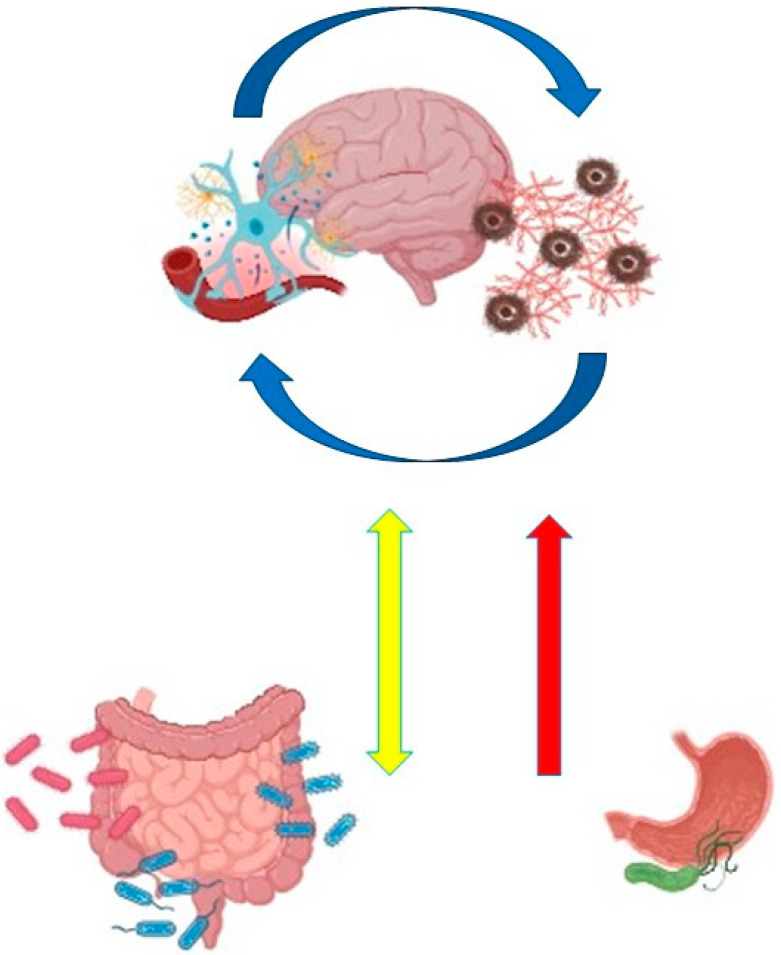
Dysbiosis-induced damage to the GI tract triggers local inflammation and compromises the intestinal barrier, exposing the ENS to chronic inflammatory insults. This promotes α-synuclein misfolding, which propagates to the brain via the vagus nerve. Concurrently, chronic GI inflammation leads to the systemic release of inflammatory mediators (single-headed red arrow), weakening the blood–brain barrier. This allows the passage of proinflammatory cytokines, immune cells, antibodies, toxins, and antigens into the central nervous system (CNS), activating microglia and driving neuroinflammation. Microglial activation exacerbates neurodegeneration within the CNS. The double-headed yellow arrow represents the bidirectional communication pathway between the GI tract and the CNS, with the vagus nerve serving as the primary conduit. A self-perpetuating cycle ensues, wherein early alterations in GI motility (from the stomach to the colon), commonly observed in PD, exacerbate dysbiosis, further contributing, as mentioned, to PD pathogenesis.

Nishiwaki et al. recently published a meta-analysis of the shotgun sequencing of the GI microbiota in Parkinson’s disease across six datasets (Japan, USA, Germany, Taiwan, and China) [24]. *Akkermansia muciniphila* counts was significantly increased while *Roseburia intestinalis* and *Faecalibacterium prausnitzii* counts were significantly decreased in PD, even after adjusting for confounding factors. PD was associated with a significant decrease in riboflavin (vitamin B2) and biotin (vitamin B7) metabolism. The reduced gut production of riboflavin may contribute to PD by exacerbating oxidative stress, mitochondrial dysfunction, and neuroinflammation [43]. Supplementation with high doses of riboflavin has been shown to improve motor deficits in PD patients [44]. Biotin possesses anti-inflammatory properties [45]. The decreased fecal biosynthesis of riboflavin and biotin was correlated with decreased fecal concentrations of polyamines (putrescine, spermidine, and spermine) and SCFAs (acetate, propionate, and butyrate). Polyamine deficiency may also play a role in PD, similar to SCFAs, as polyamines contribute to the production of the intestinal mucus layer and are involved in macrophage polarization alongside SCFAs [46,47]. Figure 1 summarizes the plausible mechanisms linking GI microbiota dysbiosis to PD.

### 5.2. Modulating Gut Microbiota Dysbiosis in PD

Different interventions have been used to restore a “healthy” GI microflora. The most effective approaches include FMT, probiotics, prebiotics, and synbiotics.

In vivo murine models of PD have demonstrated the efficacy of gut-targeted interventions in reducing dopaminergic cell loss, improving motor function, and modulating neuroinflammatory markers. A recent systematic review by Panaitescu et al., encompassing 29 studies, revealed a significant reduction in dopaminergic cell loss (82.8%, 95% CI 64.2–94.1%), microglial activation (87.5%, 95% CI 61.6–98.4%), and astrocytic activation (84.6%, 95% CI 54.5–98.1%) following these interventions (FMT in eight studies; probiotics in twenty-one studies) [48]. Additionally, the majority of studies reported improved performance in behavioral motor tests (96.4%, 95% CI 81.6–99.9%). Many studies also indicated a reduction in the prevalence of pro-inflammatory cytokines, particularly TNF-α, IL-6, and IL-1 [48].

Studies evaluating the effects of gut microbiota modulation in PD patients have also yielded promising results.

#### 5.2.1. Fecal Microbiota Transplantation

FMT is a therapeutic intervention involving the transfer of fecal material from a healthy donor to a recipient, aiming to restore the balance of the intestinal microbiota. Three studies on the effect of FMT on PD are worth mentioning [49].

In a placebo-controlled RCT by DuPont et al., conducted on 12 patients with mild-to-moderate PD and constipation, FMT through repeated doses of lyophilized products administered orally was associated with the temporary improvement of objective motor findings and subjective symptom improvements compared to the baseline. Constipation, gut transit times, and gut motility index (*p* = 0.0374) significantly improved in the FMT group. Adverse events were mild and not different between groups [50].

In the GUT-PARFECT trial, the authors assessed the clinical effects and safety of a single nasojejunal FMT in patients with mild-to-moderate PD. This placebo-controlled RCT enrolled 47 patients, of whom 43 completed all visits (21 in the treatment group, 22 in the placebo group). Radiopaque pellets test showed the significant improvement of constipation 3–6 months after FMT while the greatest improvement in motor symptoms (MDS-UPDRS motor score) was observed in the 6-to-12-month interval (mean of 5.8 points in the treatment group versus 2.7 points in the placebo group, *p* = 0.0235). This suggests a primary beneficial effect of FMT at the GI level before neurological effects become apparent. After 12 months, the MDS-UPDRS motor score significantly improved in the healthy donor group with respect to the placebo group (95% CI −11.4 to −0.2 versus −8.3 to 2.9, respectively). No major adverse events were observed and no differences were observed between the two groups [51].

Different results came from the placebo-controlled RCT conducted in Finland by Scheperjans et al. In this study, 45 patients with mild-to-moderate PD were enrolled (30 in the FMT group and 15 in the placebo group). MDS-UPDRS motor scores did not differ between the groups. Gastrointestinal adverse events, although mild, were more frequent in the FMT group (*p* = 0.003) [52].

#### 5.2.2. Prebiotics, Probiotics, and Synbiotics

Probiotics are live microorganisms that, when administered in adequate amounts, confer health benefits on the host. Prebiotics are substrates selectively utilized by host microorganisms to provide health benefits. Both probiotics and prebiotics are valuable tools for restoring a healthy gut microbiota [53].

Recent years have seen numerous studies exploring the effects of probiotics, prebiotics, and synbiotics (combinations of both) in patients with PD. The majority of studies (eleven) have focused on probiotics alone, with seven of these being placebo-controlled RCTs (Table 1). Synbiotics have been investigated in three studies (two RCTs, Table 2) while prebiotics have been evaluated in two trials (Table 3).

Most studies on probiotics alone (eleven) have originated from Eastern countries, primarily Iran (four), China/Taiwan (four), and Malaysia (one). Sample sizes have ranged from 25 to 128 PD patients. Eight trials have evaluated various probiotic combinations, predominantly *Lactobacillus* and *Bifidobacterium* species and strains, at different CFU counts. Four studies have assessed single strains: *Lactobacillus casei Shirota* (two studies), *Lactobacillus plantarum PS128*, and *Bifidobacterium animalis subsp. lactis Probio-M8*. Treatment durations have ranged from 4 to 12 weeks. Despite variations in patient characteristics and outcome measures (e.g., GI symptoms, other PD-related symptoms), all studies have reported significant improvements in motor or non-motor PD symptoms.

Regarding synbiotics, two studies were conducted in Italy and one in Malaysia. Sample sizes ranged from 30 to 120 patients. Two trials evaluated combinations of probiotics (primarily *Lactobacillus* and *Bifidobacterium*) with fructooligosaccharides (FOSs). The third trial assessed the effects of *Lacticaseibacillus paracasei DG* and inulin. All three studies reported significant improvements in GI symptoms but not in motor symptoms.

A recent meta-analysis by Mi Park et al. synthesized the results of trials assessing the effects of probiotics (alone or in combination with prebiotics) on PD patients [68]. It revealed high-quality evidence for improvements in the UPDRS Part III motor scale (SMD −0.65; 95% CI −1.11 to −0.19), non-motor symptoms (SMD −0.81; 95% CI −1.12 to −0.51), and the depression scale (SMD −0.70; 95% CI −0.93 to −0.46). Moderate-to-low-quality evidence supported significant improvements in GI motility (SMD 0.83; 95% CI 0.45–1.10), the quality of life (SMD −1.02; 95% CI −1.66 to −0.37), and the anxiety scale (SMD −0.72; 95% CI −1.10 to −0.35) [68].

**Table 3:** Main characteristics and results of principal studies on prebiotics in PD patients.

Study, Design	Sample, Patients’ Characteristics	Treatment Characteristics	Clinical Effects of Treatment	Data on Gut Microbiota or Other Relevant Findings
Becker et al., 2020 Germany [69] Three-arms open-label clinical trial	87 subjects (57 PD patients, 30 controls) Group 1: PD patients treated with prebiotics (M 18), 64.5 (42–84) y Group 2: controls treated with prebiotics (12), 61.5 (40–76) y Group 3: PD patients, no treatment (M 13), 66 (47–80) y	Group 1 and group 2: resistant starch (5 g bid) Group 3: dietary instructions alone Duration: 8 weeks	Significant improvement in non-motor and depressive symptoms at 8 weeks was noted only in group 1; effect on motor symptoms was not reported No significant change in bowel habits was noted	No significant change in gut microbiota after treatment with prebiotics was noted Significant increase in fecal butyrate concentrations and significant reduction in fecal calprotectin after treatment with prebiotics were noted in PD patients
Hall et al., 2023 USA [70] Open-label clinical trial	20 PD patients 10 PD patients medically naive (M 5), 62.9 ± 6.9 y 10 PD patients already under treatment (M 6), 65.7 ± 9.3 y	Bars containing resistant starch, rice brain, resistant maltodextrin, and inulin for 10 days (one bar = 10 g fiber) One bar in the first 3 days, then 2 bars for an additional 7 days. Duration: 10 days	Significant improvement in total GI symptom severity score was noted after prebiotic treatment Effect on MDS-UPDRS scores was not assessed	Significant reduction in levels of pro-inflammatory bacteria (e.g., *Proteobacteria*) and increase in number of SCFA-producing bacteria (e.g., *Fusicatenibacter saccharivorans, Parabacteroides merdae*) were noted Significant increase in SCFA levels and significant reductions in fecal calprotectin (intestinal inflammation), zonulin (putative marker of intestinal barrier dysfunction/inflammation), and NfL (marker of neurodegeneration) were noted

Only two studies assessed the effects of prebiotics on PD patients, one from Germany and the other from the USA. Neither study evaluated motor symptoms, but both demonstrated efficacy in improving non-motor symptoms, particularly GI issues.

Interestingly, modulating the gut microbiota through probiotics, prebiotics, or synbiotics can influence not only PD symptoms but also secondary outcomes. These include the increased production of SCFAs by gut bacteria and reduced inflammation, as evidenced by lower levels of CRP, calprotectin, or pro-inflammatory cytokines.

### 5.3. Helicobacter Pylori

HP is a Gram-negative, microaerophilic, flagellated bacterium that colonizes the human gastric mucosa in nearly half the world’s population. Typically acquired in childhood, HP persists lifelong unless eradicated [19]. While approximately 80% of infected individuals remain asymptomatic, chronic mucosal inflammation invariably develops [19]. Bacterial virulence, host genetics, and environmental factors influence the severity of infection, leading to conditions such as peptic ulcer disease, gastric cancer, and gastric MALT lymphoma [71,72,73]. HP infection has been linked to various extra-gastric diseases including iron-deficiency anemia, idiopathic thrombocytopenic purpura, ischemic heart disease, hepatobiliary disorders, and neurological conditions like migraine and ischemic stroke [19,74,75].

As early as 1965, Strang noted a higher incidence of previous gastric and duodenal ulcers in PD patients compared to controls. Moreover, PD onset was significantly earlier in those with a history of ulceration [76]. Nielsen et al. reported 23% and 45% increased risks of PD associated with proton pump inhibitor and HP eradication drug prescriptions, respectively, five years prior to diagnosis [77].

In a recent meta-analysis by Shen et al. (eight studies, 33,125 participants), the pooled OR of PD in HP-positive individuals was 1.59 (95% confidence interval, CI: 1.37–1.85) when compared with HP-negative subjects [78]. These data suggest that HP infection might represent a risk factor for PD. Additionally, HP positivity correlated with PD severity, as evidenced by higher UPDRS scores in infected patients [79].

After a careful search of the available evidence, we found a total of seven studies that evaluated the effects of HP eradication on the clinical status of PD. Table 4 summarizes their main characteristics and results. Three were double-blind placebo-controlled randomized trials. In the study (2006) by Pierantozzi et al., 34 PD patients with motor fluctuations and HP infection were randomized to eradication or placebo. Successful eradication (88.2%) was associated with long-lasting (3 months) significant improvements in clinical disability and a prolonged “on-time” duration. Interestingly, parallel to the clinical improvement, both L-dopa absorption and gastroduodenal inflammation improved significantly [80].

In 2010, another small RCT (with 30 patients) was published. Dobbs et al. found that bradykinesia improved significantly after HP eradication and was significantly worse in eradication failures (4/15, 26.7%) than in successful eradications (11/15, 77.3%). Symptom amelioration persisted at a 3.4-year follow-up. All four eradication failures were positive for ANA, suggesting a potential link between ANA positivity and a poorer response to eradication therapy [81].

In the study by Tan et al. (2020) on 67 patients with PD and HP infection, 81.3% of the 32 patients in the treatment group were successfully eradicated. However, HP eradication did not improve MDS-UPDRS motor scores at week 12 compared to placebo [82].

All open-label studies showed significant improvements in PD symptom scores in successfully eradicated patients with respect to non-eradicated patients [83,84,85,86]. Interestingly, HP disappearance was associated with parameters suggesting a link between HP and the impaired or delayed absorption of L-dopa, such as in terms of the mean L-dopa onset time or mean ON duration time.

Several pathophysiological mechanisms have been proposed to explain this potential association. L-dopa remains the cornerstone drug for the treatment of PD, with approximately 30% of the administered dose being absorbed. As demonstrated in the study by Pierantozzi et al., HP eradication is associated with a significant increase in L-dopa absorption and a reduction in gastritis/duodenitis scores [80]. L-dopa absorption is highly variable and depends on multiple factors, among which gastric emptying and pH play critical roles. Gastroduodenal damage caused by HP infection may alter both of these parameters, significantly contributing to reduced L-dopa absorption [87].

Moreover, an in vitro interaction between L-dopa and HP bacterial adhesins has been demonstrated, suggesting an additional mechanism for reduced L-dopa absorption in HP-positive patients [88]. HP can also utilize L-dopa in vitro as a growth substrate under iron-restricted conditions, converting it into an amino acid beneficial for its metabolism [89].

Other plausible mechanisms, similar to those proposed for dysbiosis in general, include the chronic inflammatory response induced by HP infection. Beginning in the stomach, this inflammatory response can become systemic, potentially crossing the blood–brain barrier, which may be more permeable due to systemic inflammation. Once in the CNS, toxins, immune cells, antibodies, or antigens could activate microglia, inducing neuroinflammation [9,16]. Cross-reactivity between HP and host antigens has also been documented, potentially leading to autoantibody production. This may explain the association between HP infection and various autoimmune diseases [90]. The presence of autoantibodies in PD patients has been corroborated by the study conducted by Dobbs et al., as previously mentioned [82]. Additionally, Suwarnalata et al. detected elevated autoantibodies against proteins essential for normal neurological function almost exclusively in HP-positive PD patients. These antibodies targeted nuclear factor I subtype A, platelet-derived growth factor B, and eukaryotic translation initiation factor 4A3 [91]. Chronic HP infection may, therefore, trigger an autoimmune response, potentially activating the brain’s immune system and causing neuronal damage through neuroinflammation [27].

Finally, HP may contribute to PD by disrupting gastrointestinal microbiota [92,93,94]. This dysregulation could result from bacterial virulence factors such as CagA and VacA, varying degrees of gastric damage, dietary modifications, pH changes induced by HP-related gastric damage, or the frequent use of PPIs in patients with HP-related gastritis. Alterations in microbiota composition, including reduced *Bacteroidetes* and increased *Firmicutes* and *Proteobacteria* levels, have been observed in HP-gastritis patients compared to healthy individuals [95].

### 5.4. Small-Intestinal Bacterial Overgrowth

SIBO is a distinct form of dysbiosis characterized by a quantitative (excessive bacterial growth) and/or qualitative (altered bacterial composition) imbalance in the small intestine [20,21]. The dysregulation of gut flora leads to various GI symptoms, the most common of which are bloating, abdominal distension, and diarrhea. Nutritional deficiencies and significant weight loss may also occur, particularly in elderly individuals [20,21].

The gold standard for SIBO diagnosis is a small bowel jejunal aspirate showing ≥10⁵ CFU/mL. However, based on evidence from per-endoscopic aspirate studies, a 2017 consensus revised the diagnostic threshold to ≥10^3^ CFU/mL [96]. Despite this adjustment, the clinical application of jejunal aspirate remains limited due to its invasive nature, high cost, and restricted availability. As a result, GBT and LBT have gained popularity in clinical practice due to their non-invasive nature, accessibility, and lower cost [96,97]. Both tests demonstrate acceptable sensitivity and specificity for clinical and research purposes [96,97]. GBT is considered more accurate than LBT because of its higher specificity. However, due to the properties of the substrates used, GBT is more effective for detecting SIBO in the upper portion of the small intestine while LBT is better suited for identifying SIBO in the distal segments [21]. Therefore, combining both tests may improve diagnostic accuracy [21].

Risk factors for SIBO include various anatomical abnormalities or postsurgical structural changes, hypochlorhydria resulting from gastric surgery, atrophic gastritis, the use of PPIs, and intestinal hypo- or dysmotility caused by medications or systemic and intestinal diseases such as diabetes or IBS [21,98]. Chronic HP gastritis, which is associated with reduced gastric acidity and secondary motility impairment, may represent a predisposing factor for the development of SIBO in the distal digestive tract.

A recent systematic review and meta-analysis by Liao et al. synthesized data from eight studies, including 874 patients, to investigate this association. The analysis revealed that HP infection was associated with significantly increased odds of SIBO (OR 1.82, 95% CI 1.29–2.58, *p* < 0.001) [99]. Notably, these findings were consistent across variations in the study location, patient comorbidities, exposure to PPIs, and the methods used to assess HP infection and SIBO. These results highlight the importance of evaluating SIBO in patients with gastrointestinal symptoms and HP infection [21,99].

Gastrointestinal motility dysfunction, characterized primarily by dysphagia, gastric emptying disorders, and constipation, is a hallmark of PD and often precedes its clinical diagnosis by several years [3]. These motility alterations may promote the development of SIBO in PD patients.

Since 1996, numerous studies have examined the prevalence of SIBO and the effects of its eradication in individuals with PD. A systematic review and meta-analysis conducted by Li et al. provided a comprehensive summary of the available literature on SIBO prevalence in PD patients up to 2020 [100]. The analysis included 11 studies involving 973 participants, reporting a pooled SIBO prevalence of 46% (95% CI 36–56). The prevalence was higher in patients from Western countries (52%, 95% CI 40–64) compared to those from Eastern countries (33%, 95% CI 22–43). The pooled odds ratio (OR) for SIBO in PD patients compared with healthy controls was 5.22 (95% CI 3.33–8.19, *p* < 0.00001), indicating a significantly increased risk. Interestingly, SIBO prevalence was higher in studies using LBT (51%, 95% CI 37–65) compared to those using GBT (35%, 95% CI 20–50). The highest prevalence was observed in studies employing both LBT and GBT for diagnosis (55%, 95% CI 38–72) [100].

Since the publication of the 2020 meta-analysis, only two original studies have specifically evaluated the presence of SIBO in PD patients. Kuai et al. assessed SIBO using the LBT in 11 PD patients, all of whom tested positive at enrollment [101]. Zhou et al. conducted a larger study enrolling 70 PD patients (35 with and 35 without mild cognitive impairment) and 17 healthy controls matched for sex and age. All participants underwent hydrogen and methane LBT. The study found that hydrogen levels, both alone and in combination with methane, were significantly higher in PD patients with mild cognitive impairment (who also exhibited a greater PD symptom burden) compared to those without cognitive impairment and healthy controls [102]. A positive methane breath test is indicative of a specific form of SIBO known as intestinal methanogen overgrowth, caused by *Archaea*—most commonly, *Methanobrevibacter smithii*. This organism has been associated with delayed small bowel and colonic transit times, contributing to constipation [103]. Since constipation is a prevalent symptom in PD, Zhou et al.’s findings suggest that testing for both hydrogen and methane could be valuable in identifying SIBO in PD patients.

To the best of our knowledge, only two studies have evaluated the effects of SIBO eradication on PD symptoms (Table 5). Fasano et al. treated 18 PD patients with motor fluctuations and a diagnosis of SIBO (based on positivity to either GBT or LBT) using rifaximin (1200 mg/day for 7 days). Successful SIBO eradication was associated with a significant improvement in motor fluctuations without affecting the pharmacokinetics of L-dopa. No significant side effects were reported [104].

In the study by Kuai et al., 11 PD patients with positive LBT underwent FMT. Following treatment, LBT normalized in all patients, accompanied by a significant improvement in the orocecal transit time. Additionally, after FMT, scores for the H-Y stage, UPDRS, NMSS, PAC-QOL, and Wexner constipation scale showed significant reductions. No significant side effects were reported [101].

SIBO recurrence is common, particularly when predisposing conditions cannot be eliminated. Recurrence rates of 27.5% and 43.7% have been observed at 6 and 9 months, respectively, following successful rifaximin treatment [105]. A similarly high recurrence rate was reported in PD patients 6 months after successful antibiotic therapy [104]. This is unsurprising given the well-documented and prevalent intestinal dysmotility associated with PD.

The pathophysiological mechanisms proposed to explain the association between SIBO and PD are similar to those described for dysbiosis in general.

## 6. Conclusions

In recent years, several review articles have explored the role of the gut microbiota in neurological diseases with a particular focus on PD. The unique contribution of our work lies in providing a comprehensive, up-to-date, and critical review of the current knowledge, also highlighting the existing gaps in the evidence regarding the etiopathogenetic role of the GI microbiota and the therapeutic potential of its modulation in PD. In a single article, we have collected the available literature not only on dysbiosis in general but also on two specific and highly relevant forms: gastric dysbiosis caused by HP and the one affecting the small intestine, namely SIBO.

The GI system is closely interconnected with the CNS through the so-called gut–brain axis, with the vagus nerve being the primary communication pathway. Today, the term ‘microbiota–gut–brain axis’ is more appropriate as the GI microbiota functions as an organ within an organ, profoundly influencing the CNS through various mechanisms. In the case of dysbiosis, a “diseased” microbiota negatively impacts this axis and may play a significant role in the pathogenesis of PD.

The existing literature demonstrates that the intestinal microbiota in PD patients is altered compared to that in controls without PD. Two specific forms of GI dysbiosis are commonly observed in PD patients: gastric HP infection and SIBO. Both conditions have a significantly higher prevalence in PD compared to controls. HP eradication appears effective in alleviating both GI and motor symptoms of PD while SIBO decontamination may also improve PD symptoms, though the limited data available have not provided solid evidence. As suggested by studies on SIBO, the recurrence of dysbiosis is highly frequent in PD patients.

Animal studies on PD models have shown that modulating the microbiota through FMT or probiotics/prebiotics can improve symptoms, reduce neurodegeneration, and mitigate inflammation. Human studies have corroborated these findings, particularly for probiotics, which have been shown to alleviate not only GI symptoms in PD patients but also motor symptoms and inflammation. However, as with SIBO, the risk of dysbiosis recurrence following treatment is likely high. This suggests that cyclic treatments might be beneficial for patients predisposed to relapse, such as those with PD.

The role of the GI microbiota in the pathogenesis of neurological disorders like PD is evident. However, clinical trials using probiotics, prebiotics, FMT, and antibiotics to modulate dysbiosis in PD have yielded low-quality data due to methodological limitations. Most trials are small, with outcomes that are not consistently comparable. Treatments vary widely, involving different combinations and treatment durations.

Future research should prioritize large-scale RCTs, ideally multicenter studies, to increase sample sizes. In particular, regarding probiotic interventions, future studies should prioritize the standardization of probiotic strains, dosages, and treatment durations to enhance the comparability and reliability of results. Additionally, they should assess the intestinal microbiome and associated metabolic alterations before and after treatment and include the longitudinal monitoring of the microbiome to determine the frequency of dysbiosis recurrence and the efficacy of repeated treatments in preventing relapse. This approach could provide stronger evidence and pave the way for tailored interventions targeting microbiota modulation in PD.

## Figures and Tables

**Table 1:** Main characteristics and results of principal studies on probiotics in PD patients.

Study, Design	Sample, Patients’ Characteristics	Treatment Characteristics	Clinical Effects of Treatment	Data on Gut Microbiota or Other Relevant Findings
Cassani et al., 2011 Italy [54] Open-label single-arm trial	40 PD patients with functional constipation	Dietetic therapy for constipation + Fermented milk drink—65 mL—containing 6.5 × 10^9^ CFU of *Lactobacillus casei Shirota* Duration: 5 weeks	Significant increase in the number of days per week in which stools were of normal consistency and significant reductions in the number of days per week in which patients felt bloated and experienced abdominal pain and sensation of incomplete emptying Effect of MDS-UPDRS scores not assessed	No data on gut microbiota
Georgescu et al., 2016 Romania [55] Open-label RCT	40 PD patients Group 1: 20 patients (M 7), 75.7 ± 9.7 y Group 2: 20 patients (M 10), 69.8 ± 5.6 y Age significantly different between the groups	Group 1: trimebutine 200 mg tid Group 2: *Lactobacillus acidophilus* and *Bifidobacterium infantis*, 2 tablets each 60 mg All patients: increased fluid intake to 2 L/d and dietary fibers 20–25 g/d Duration: 12 weeks	Significant improvement in all non-motor GI symptoms after treatment in the first group; significant improvement in abdominal pain and bloating but not in constipation in the second group Effect on MDS-UPDRS scores was not reported	No data on gut microbiota
Tamtaji et al., 2018 Iran [56] Double blind placebo-controlled RCT	60 PD patients Treatment group: 30 patients, 68.2 ± 7.8 y Placebo group: 30 patients, age 67.7 ± 10.2 y Sex not reported	Probiotics: *Lactobacillus acidophilus, Bifidobacterium bifidum, Lactobacillus reuteri*, and *Lactobacillus fermentum* (each 2 × 10^9^ CFU/g) Duration: 12 weeks	Significant improvement in MDS-UPDRS total score in the treatment group Effects on GI symptoms were not reported	No data on gut microbiota Significant reductions in HS-CRP, MDA, insulin levels, and insulin resistance; increase in glutathione levels and improvement of insulin sensitivity in the probiotic group
Borzabadi et al., 2018 Iran [57] Double blind placebo-controlled RCT	50 PD patients Treatment group: 25 patients (M 17), 66.9 ± 7.0 y Placebo group: 25 patients (M 16), 66.7 ± 10.7 y	Probiotics: *Lactobacillus* *acidophilus, Lactobacillus reuteri, Lactobacillus fermentum,* and *Bifidobacterium bifidum* (each 2 × 10^9^ CFU) Duration: 12 weeks	Effects on PD symptoms were not reported	No data on gut microbiota Significant reductions in expression of genes related to inflammation, such as IL-1, IL-8, TNF-α, and TGF-β, in the probiotic group
Tan et al., 2021 Malaysia [58] Double blind placebo-controlled RCT	72 PD patients with functional constipation Treatment group: 34 patients (M 20), 70.9 ± 6.6 y Placebo group: 38 patients (M 28), 68.6 ± 6.7 y	*Lactobacillus acidophilus, Lactobacillus reuteri, Lactobacillus gasseri, Lactobacillus rhamnosus, Bifidobacterium bifidum, Bifidobacterium longum, Enterococcus faecalis,* and *Enterococcus faecium*, 10 × 10^9^ CFU Duration: 4 weeks	Significant improvement in SBM, stool consistency, constipation severity score, and quality of life related to constipation in treatment group Effect on MDS-UPDRS scores was not assessed	No data on gut microbiota Changes in fecal calprotectin from baseline to end of intervention were not significantly different between groups
Lu et al., 2021 Taiwan [59] Open-label single-arm baseline-controlled trial	25 PD patients (M 17), 61.8 ± 5.7 y	*Lactobacillus plantarum* *PS128*, 60 × 10^9^ CFU Duration: 12 weeks	Significant improvement in UPDR motor scores in both the OFF and ON states, the duration of the ON period, and the quality of life after treatment No significant change was noted in GI symptoms	No data on gut microbiota Significant decrease in markers of oxidative damage (plasma myeloperoxidase and urinary 8-hydroxy-2′-deoxyguanosine)
Du et al., 2022 China [60] Open-label RCT	46 PD patients with Constipation Treatment group: 23 patients (16 M), 68.4 ± 7.6 y 23 controls (10 M), 66.7 ± 8.7 y	*Bacillus licheniformis* (2.5 × 10^9^ CFU/capsule, 2 capsules tid) + *Lactobacillus acidophilus,* *Bifidobacterium longum* and *Enterococcus faecalis* (1 × 10^7^ CFU per strain), 4 capsules bid Duration: 12 weeks	Significant improvement in constipation in the treatment group (average number of complete bowel movements per week, BSS score, PAC-SYM score, PAC-QOL score, degree of defecation effort score) Effect on MDS-UPDRS scores was not assessed	After treatment with probiotics, *Christensenella Marseille-P2437* levels significantly increased and *Eubacterium oxidoreducens, Eubacterium_hallii* and *Odoribacter N54.MGS-14* levels decreased
Sun et al., 2022 China [61] Double blind placebo-controlled RCT	82 PD patients Treatment group: 48 patients (M 32), 66.5 ± 7.0 y Placebo group: 34 patients (M 23), 68.8 ± 6.9 y	*Bifidobacterium animalis subsp. lactis Probio-M8*, 3 × 10^10^ CFU/day) Duration: 12 weeks	Significant improvement in motor symptoms, sleep quality, anxiety state, mental state, and depression in treatment group was noted Significant improvement in GI symptoms (BSS, PAC-QOL, times of spontaneous defecations and completed defecations per week, feces hardness, and difficulty in defecation)	Significantly more species-level genome bins of *Bifidobacterium animalis, Ruminococcaceae, and Lachnospira* and less *Lactobacillus fermentum* and *Klebsiella oxytoca* were noted in the probiotic group Treatment with probiotics was associated with significant increase in number of species involved in tryptophan degradation, GABA, SCFAs, and secondary bile acid biosynthesis, as well as serum acetic acid and dopamine levels
Ghalandari et al., 2023 Iran [62] Triple-blind, parallel RCT	27 PD patients Treatment group: 14 (M 8), 68.0 ± 6.7 y Placebo group: 13 (M 7), 68.5 ± 6.9 y	*Lactobacillus* *plantarum, Lactobacillus* *casei, Lactobacillus* *acidophilus, Lactobacillus* *bulgaricus; Bifidobacterium infantis, Bifidobacterium Longum, Bifidobacterium breve; Streptococcus thermophilus* (each genus accounting for 1.5 × 10^11^ CFU) Duration: 8 weeks	Significant improvement in frequency of bowel movements and stool consistency was noted in treatment group No significant differences in PD motor symptoms were noted	No data on gut microbiota
Yang et al., 2023 China [63] Double blind placebo-controlled RCT	128 PD patients Treatment group: 65 patients (M 31), 67.2 ± 6.5 y Placebo group: 63 patients (M 42), 69.6 ± 6.4 y	Fermented milk containing 1 × 10^10^ living cells of *Lacticaseibacillus strain Shirota* Duration: 12 weeks	Significant improvement in constipation-related symptoms (Wexner score, BSS score, BMs, PAC-QOL) and significant reduction in use of laxatives were noted in treatment group Significant improvement in non-motor symptoms (NMSS, HAMD-17, and HAMA) was noted in treatment group Significant improvement in QL scores (PDQ-39) was noted in treatment group	No changes in the global gut microbiome were noted after intervention, but significantly increased abundance of the genus *Lacticaseibacillus* in the probiotic group compared with baseline and placebo group was noted Fecal concentration of L-tyrosine significantly decreased and plasma concentration of L-tyrosine increased in probiotic group
Zali et al., 2024 Iran [64] Double blind placebo-controlled RCT	46 PD patients Treatment group: 23 patients (M 14), 56.3 ± 10.2 y Placebo group: 23 patients (M 15), 55.7 ± 11.0 y	*Lactobacillus* *acidophilus, Lactobacillus* *rhamnosus, Lactobacillus* *reuteri, Lactobacillus* *paracasei; Bifidobacterium longum; Bacillus coagulans* (2 × 10^9^ CFU) + 400 IU vitamin D Duration: 12 weeks	Significant improvements in anxiety, GI symptom rating scale, and UPDRS sub-scales I, III, and IV were noted in treatment group	Significant decrease in IL-1β, INF-γ, IL-6, and MDA levels and significant increase in IL-10 levels were noted in the group treated with probiotics (and vitamin D)

**Table 2:** Main characteristics and results of principal studies on synbiotics in PD patients.

Study, Design	Sample, Patients’ Characteristics	Treatment Characteristics	Clinical Effects of Treatment	Data on Gut Microbiota or Other Relevant Findings
Barichella et al., 2016 Italy [65] Double-blind placebo-controlled RCT	120 PD patients Treatment group: 80 patients (M 41), 71.8 ± 7.7 y Placebo group: 40 patients (M 24), 69.5 ± 10.3 y	Fermented milk with the following: -Probiotics: *Streptococcus salivarius subsp thermophilus; Enterococcus faecium; Lactobacillus rhamnosus GG, acidophilus, plantarum, paracasei, delbrueckii subsp* *bulgaricus; Bifidobacterium breve* and *animalis subsp lactis* (total content of probiotics: 250 × 10^9^ CFU) + -Prebiotics: FOS 2% Duration: 4 weeks	Significant improvement in constipation (increase in the number of complete bowel movements) Effect on MDS-UPDRS scores not assessed	No data on gut microbiota
Ibrahim et al., 2020 Malaysia [66] Double blind placebo-controlled RCT	55 PD patients with functional constipation Treatment group: 27 patients (M 16), 69.0 (64.0–74.0) y Placebo group: 28 patients (M 17), 70.5 (62.0–70.3) y	-Probiotics: *Lactobacillus acidophilus 107 mg, Lactobacillus casei 107 mg, Lactobacillus lactis 107* *mg, Bifidobacterium infantis 107 mg,* and *Bifidobacterium* *longum 107 mg* + -Prebiotics: FOS 2% Duration: 8 weeks	Improvement in constipation in treatment group (significant improvement of BOF and GTT, and reduction % of patients remaining constipated) No significant differences in the total MDS-UPDRS score, NMSS scores and PDQ-39 scores between groups	No data on gut microbiota
Andreozzi et al., 2024 Italy [67] Open-label single-arm trial	30 PD (M 20) 64.7 ± 7.1 y patients with functional constipation	Probiotics: *Lacticaseibacillus paracasei DG* (≥8 × 10^9^ CFU) Prebiotics: fiber inulin 4.0 g Duration: 12 weeks	No significant improvement in motor symptoms (MDS-UPRDS part 3) Significant improvement in: -non-motor symptoms (MDS-UPDRS part 1 and anxiety, depression and autonomic dysfunction scores) -constipation (PAC-SYM score, number of complete bowel movement and BSFS)	Significant increase in abundance of the order *Oscillospirales*, family *Oscillospiraceae,* and species *Faecalibacterium prausnitzii* was noted after treatment

**Table 4:** Main characteristics and results of principal studies on HP eradication in PD patients.

Study, Design	Sample, Patients’ Characteristics	Treatment Characteristics	Clinical Effects of Treatment	Other Relevant Findings
Pierantozzi et al., 2006 Italy [80] Double blind placebo-controlled parallel-group RCT	34 PD patients with motor fluctuations—HP infection and eradication assessed by gastric biopsy Eradication group 17 patients (M 8), 64.9 ± 9.6 y Placebo group 17 patients (M 8), 66.3 ± 6.9 y	Eradication therapy: omeprazole 20 mg BID, amoxicillin 1 g BID, clarithromycin 500 mg BID, 7 days Allopurinol (chosen for its antioxidant properties) 100 mg BID, 15 days Placebo and active therapies were supplied and formulated in the same way	Eradication group: 2 still HP-positive Placebo group: all HP-positive HP eradication but not allopurinol was associated with significant improvement in clinical disability and a prolonged “on-time” duration	HP eradication, but not allopurinol, was associated with significant increase in L-dopa absorption Gastritis/duodenitis scores significantly decreased in line with a better L-dopa pharmacokinetics
Yong Lee et al., 2008 South Korea [81] Open-label study	65 PD patients with motor fluctuations and HP infection HP infection and eradication assessed by UBT	Eradication therapy: esomeprazole 20 mg BID, amoxicillin 500 mg BID, clarithromycin 500 mg BID, 7 days	Eradicated: 35 patients (M 20), 60.0 ± 9.5 y Not eradicated: 30 patients (M 16), 60.2 ± 8.4 y Delay to L-dopa ‘‘onset’’ time was significantly greater and ‘‘on-time’’ duration shorter in the infected than in noninfected subjects Delay in L-dopa ‘onset’ time was significantly reduced and ‘on-time’ duration significantly prolonged after successful eradication	-
Dobbs et al., 2010 United Kingdom [82] Double blind placebo-controlled RCT	30 PD patients with HP infection assessed by gastric biopsy. HP eradication assessed by UBT only after de-blinding Eradication group: 14 patients (M 6), 59 (41–78) y Placebo group:16 patients (M 13), 63 (45–81) y	Eradication therapy: omeprazole 20 mg BID, amoxicillin 500 mg BID, clarithromycin 500 mg BID, 7 days (metronidazole 400 mg or tetracycline 500 mg QID was used in case of in vitro insensitivity or suspected intolerance)	20 patients were de-blinded early due to marked clinical deterioration Eradication group: 4 still HP-positive Placebo group: all HP-positive Brady-hypokinesia significantly improved after successful blinded active treatment compared with placebo and significantly worse in patients with eradication failure compared with those successfully eradicated Correction of deficit continued for 3.4 years post eradication Significance was maintained also after excluding patients taking L-dopa	ANA was present in all 4 eradication failures In the remaining cases, ANA positivity associated with a significantly poorer response during the year following eradication therapy
Hashim et al., 2014 Malaysia [83] Open-label study	82 PD patients HP infection and eradication assessed by UBT	Eradication therapy: esomeprazole 40 mg BID, amoxicillin 1000 mg BID, clarithromycin 500 mg BID, 7 days	HP-positive: 27 (6 lost at follow-up); 21 (M 10) patients, 65.1 ± 10.0 y HP-negative: 55 (M 24), 67.5 ± 7.3 y All 21 HP-positive patients treated were successfully eradicated Significantly poorer total UPDRS and PDQ-39 scores were noted in HP-positive patients than in HP-negative patients Significant improvement in mean L-dopa onset time; mean ON duration time; total UPDRS scores; UPDRS scores for parts II, III and IV; total PDQ-39 scores; and subdomains of mobility, ADL, emotional well-being, and stigma were noted 12 weeks post eradication	-
Liu et al., 2017 China [84] Open-label study	48 PD patients HP infection and eradication assessed by UBT	Eradication therapy: omeprazole 20 mg BID, amoxicillin 1 g BID, clarithromycin 500 mg BID, 14 days	Group 1: HP-negative; 26 patients (M 14), 63.7 ± 8.3 y Group 2: HP-positive refusing eradication therapy; 12 patients (M 4), 62.7 ± 10.0 y Group 3: HP-positive receiving eradication therapy; 10 patients (M 5), 63.2 ± 7.4 y UPDRS-III scores were significantly lower compared to baseline at 1-year follow-up in group 3 UPDRS-26 was significantly improved in group 3 when compared to group 1 and group 2 at 1-year follow-up	-
Tan et al., 2020 Malaysia [85] Double blind placebo-controlled RCT	67 PD patients with HP infection HP infection assessed by UBT and serology HP eradication assessed by UBT	Eradication therapy: omeprazole 20 mg BID, amoxicillin 1 g BID, clarithromycin 500 mg BID, 7 days	Eradication group: 32 patients (M 19), 66.0 ± 9.8 y Placebo group: 35 patients (M 22), 67.4 ± 8.1 y Successful eradication was achieved in 81.3% of the treatment group and 9.1% with the placebo HP eradication was not associated with significant improvement in MDS-UPDRS motor scores at week 12	Lactulose breath test was used to assess SIBO SIBO status did not influence treatment results
Lolekha et al., 2021 Thailand [86] Open-label study	40 PD patients HP infection and eradication assessed by UBT	Eradication therapy: omeprazole 40 mg BID, amoxicillin 1 g BID, clarithromycin 500 mg BID, 14 days	HP-negative: 26 patients (M 14), 63.7 ± 8.3 y HP-positive: 10 patients (M 5), 63.2 ± 7.4 y Successful eradication was achieved in 77.3% of patients In successfully eradicated patients, the following were noted: - Significant decrease in daily ‘off’ time and increase in daily ‘on’ time; - Significant improvement in total wearing-off score and the GI symptom score; - No significant improvement in L-dopa onset time, UPDRS motor score, or quality of life score	-

**Table 5:** Main characteristics and results of principal studies on SIBO decontamination in PD patients.

Study, Design	Sample, Patients’ Characteristics	Treatment	Clinical Effects of Treatment	Other Relevant Findings
Fasano et al., 2012 Italy [104] Open-label study	33 PD patients (M 18), 67.8 ± 8.5 y SIBO assessed by GBT and LBT: positivity if at least one was positive SIBO positivity: 54.5% (18/33)	Decontamination therapy: rifaximin 400 mg TID for 7 days GBT/LBT repeated 1 month after the end of the treatment Decontamination rate: 77.8% (14/18)	Successful decontamination associated with significant improvement in motor fluctuations without affecting the pharmacokinetics of L-dopa	No side effects SIBO recurrence at 6-month follow-up: 42.9% (6/14)
Kuai et al., 2021 China [101] Open-label study	11 PD patients (M 7), 62.4 ± 13.1 y SIBO assessed by LBT SIBO positivity: 100% (11/11)	FMT (40–50 mL of frozen fecal microbiota transplanted into the intestine through a nasoduodenal tube) LBT repeated 12 weeks after the treatment Decontamination rate: 100% (11/11)	Significant reduction in H-Y grade, UPDRS, NMSS, PAC-QOL score, and Wexner constipation score after FMT	Increased abundance of *Blautia* and *Prevotella* and decreased abundance of *Bacteroidetes* in PD patients after FMT were noted

## Abbreviations

ADL	activity of daily living
ANA	antinuclear antibodies
BID	bis in die,’ two times a day’
BMs	number of bowel movements.
BSFS	Bristol stool form scale
BSS	Bristol stool scale
CagA	cytotoxin-associated gene A
CFU	colony-forming unit
CI	confidence interval
CNS	central nervous system
ENS	enteric nervous system
FMT	fecal microbiota transplantation
FOS	fructooligosaccharides
GABA	gamma-aminobutyric acid
GBT	glucose breath test
GI	gastrointestinal
GTT	gut transit time
HAMA	Hamilton anxiety scale
HAMD-17	Hamilton depression rating scale (HDRS)
HP	*Helicobacter pylori*
HS-CRP	high-sensitivity C-reactive protein
H-Y	Hoehn–Yahr
IBS	irritable bowel syndrome
IL	interleukin
LBT	lactulose breath tests
MALT	mucosa-associated lymphoid tissue
MDA	malondialdehyde
MDS-UPDRS	Movement Disorder Society Unified Parkinson’s Disease Rating Scale
NMSS	Non-Motor Symptoms Scale
NMSS	Non-Motor Symptoms Scale for PD
NfL	neurofilament light-chain protein
OR	odds ratio
PAC-QOL	patient assessment of constipation
PAC-SYM	patient assessment of constipation symptoms
PD	Parkinson’s disease
PDQ-39	Parkinson’s disease questionnaire
PPIs	proton pump inhibitors
QID	quater in die, ‘four times a day’
RCT	randomized controlled trial
SBM	spontaneous bowel movements
SCFA	short-chain fatty acid
SIBO	small-intestinal bacterial overgrowth
SMD	standard mean difference
TGF	transforming growth factor
TID	ter in die, ‘three times a day’
TLR	toll-like receptor
TNF	tumor necrosis factor
UBT	urea breath test
VacA	vacuolating cytotoxin A
